# Modular enhancement of circularly polarized luminescence in Pd_2_A_2_B_2_ heteroleptic cages†

**DOI:** 10.1039/d3cc00262d

**Published:** 2023-02-21

**Authors:** Jacopo Tessarolo, Elie Benchimol, Abdelaziz Jouaiti, Mir Wais Hosseini, Guido H. Clever

**Affiliations:** a Department of Chemistry and Chemical Biology, TU Dortmund University Otto-Hahn-Straße 6 Dortmund 44227 Germany guido.clever@tu-dortmund.de; b Laboratoire de Tectonique Moléculaire, UMR Unistra-CNRS 7140, Université de Strasbourg 4 rue Blaise Pascal 67070 Strasbourg France

## Abstract

Metal-mediated assembly allows us to combine an achiral emissive ligand A with different chiral ligands (such as B) in a non-statistical fashion, obtaining Pd_2_A_2_B_2_ heteroleptic cages showing circularly polarized luminescence (CPL). By using the ‘shape complementary assembly’ (SCA) strategy, the cages are exclusively obtained as *cis*-Pd_2_A_2_B_2_ stereoisomers, as confirmed by NMR, MS and DFT analyses. Their unique chiroptical properties derive from the synergy of all the building blocks. Ligand B imparts the chiral information of its aliphatic backbone, comprising two stereogenic sp^3^ carbon centres, to the overall structure, causing CD and CPL signal induction for the chromophore on ligand A. The heteroleptic cage shows CPL with a |*g*_lum_| value of 2.5 × 10^−3^, which is 3-times higher than that for a progenitor based on aromatic helical building block H, thus opening a rational route towards optimizing the CPL properties of self-assembled nanostructures in a modular way.

## Abstract 2

Two one-step-synthesized ligands, one chiral and one emissive, assemble with Pd^II^ into a heteroleptic cage that shows enhanced CPL.

## Graphical abstract

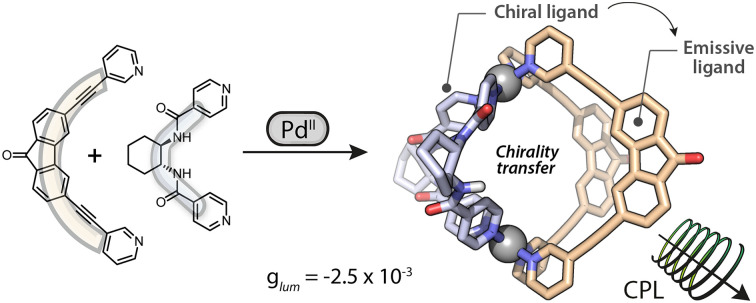

Coordination cages have seen a considerable development since the seminal works of Saalfrank,^1^ Stang,^2^ Raymond^3^ and Fujita,^4^ and others.^5–9^ Taking advantage of their nanoscale cavity and inherent plasticity, they are employed in a broad range of applications such as guest sensing,^7,10,11^ chemical separation,^12^ catalysis,^13,14^ and the development of transformation networks.^15^ Furthermore, the implementation of functional building blocks, paired with a careful structural design, allows us to widen and tailor the capabilities of such nanoscopic assemblies. Recently, the introduction of multiple functionalities within a single architecture has moved into focus. However, the combination of different building blocks (*i.e.*, ligands) within the same structure bears the risk of creating statistical distributions of multi-component assemblies (different stoichiometries, diastereomers) without proper morphological control, thus complicating the establishment of clear structure–function relationships. To overcome this problem, several rational strategies to restrict multi-component self-sorting, resulting in the exclusive formation of a single heteroleptic structure, have been developed.^16^ Suitable approaches involve, but are not limited to, shape-complementary assembly (SCA),^17–20^ coordination sphere engineering (CSE),^21–23^ control *via* steric bulk in the ligand backbone,^24^ guest templation,^25,26^ selective ligand to ligand interactions,^27^ or the use of ligands with reduced symmetry.^28–31^ The number of examples in which different functions (and not just building blocks) have been incorporated into heteroleptic cages is still very small. Also, the systematic analysis of functional unit interplay and realization of emergent properties is still in its infancy.

Among a vast choice of desirable functionalities, the combination of chiral building blocks with chromophores, such as photoswitches, dyes or luminophores, within distinct cage assemblies is of interest for applications in supramolecular recognition and catalysis or, in the latter case, for achieving nanostructures with tuneable chiroptical properties. Here, a particular focus is set on circularly polarized luminescence (CPL).^32^ The use of metal-mediated self-assembly comes in handy here, as it allows us to avoid tedious synthetic procedures otherwise necessary to covalently couple chiral groups and chromophores. Previous examples in the area of cage chemistry have shown that chirality can be transferred from guest-to-host,^33–35^ host-to-guest,^36^ or from ligand-to-ligand,^37^ with the emergence of chiroptical properties such as circular dichroism (CD) and CPL, from initially achiral building blocks.

In our previous work,^37^ we exploited the SCA strategy to combine Pd^II^ cations, enantiopure helicene ligands (H) and fluorenone-based emissive ligands (A), successfully achieving the first multifunctional *cis*-Pd_2_H_2_A_2_ heteroleptic cages showing CPL from the formerly achiral ligand component, with |*g*_lum_| values up to 9.0 × 10^−4^ (Scheme 1a). The cages with their chiroptical response were used to probe the encapsulation of a small guest molecule, resulting in a 4-fold boost of CPL intensity as well as a pronounced shift of the emission maximum. However, our previous system left a number of open questions and room for improvement: making of the helicene-based ligand requires several synthetic steps. Furthermore, it already shows CPL on its own (|*g*_lum_| = 2.8 × 10^−2^),^38^ that – alongside its emission – is lost upon coordination to Pd^II^ cations in order to create a 3-dimensional confined space. Also, the absorption bands of ligands H and A overlap, thus making it impossible to selectively excite the fluorenone chromophore alone, hence raising questions concerning the CPL mechanism. In this case, it was not readily conceivable whether CPL arises from homo/hetero-exciton coupling,^39^ or chiral energy transfer,^40,41^ or simply derives from an asymmetric distortion of the emissive ligand itself or its immediate environment. To gain more insight, we herein report the modular synthesis and analysis of the second example of a CPL-emitting Pd-based heteroleptic cage. Therefore, we used the same emissive achiral ligand (A) as in our previous system, while changing the chiral component from an aromatic to an aliphatic cyclohexane backbone, functionalized with amide linkers and *para*-pyridine donor groups (B). Ligand B is inspired by chiral cyclohexyl based tectons^42^ that have been used for the generation of chiral coordination networks in the crystalline phase.^43–45^

**Scheme 1:**
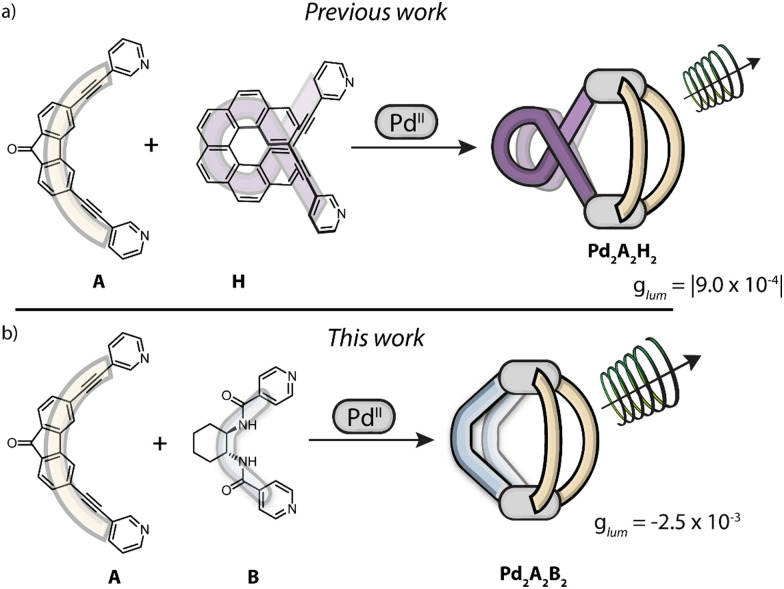
Self-assembly of Pd^II^-based heteroleptic cages displaying CPL *via* ligand-to-ligand chirality transfer. The emissive ligand A is based on a fluorenone moiety, the chiral ligands are based on (a) a helicene backbone (H) or (b) a cyclohexane backbone (B).

Both ligands A and enantiomerically pure *R*,*R*-B (from now on just B) are obtained in a one-step reaction from commercially available reagents, following literature-reported procedures.^46–49^ These simple building blocks are then combined with [Pd(MeCN)_4_](BF_4_)_2_ in acetonitrile in a 1 : 1 : 1 ratio, yielding a rather structurally complex heteroleptic cage [Pd_2_A_2_B_2_](BF_4_)_4_ (namely Pd_2_A_2_B_2_; Scheme 1b). The assembly forms virtually quantitatively, requires no purification and its structure was confirmed by a number of analytical results, including ^1^H-NMR, where we observe a typical downfield shift of protons H_a_ and H_1_ of the pyridine donors of A and B, respectively (Fig. 1a).

**Fig. 1:**
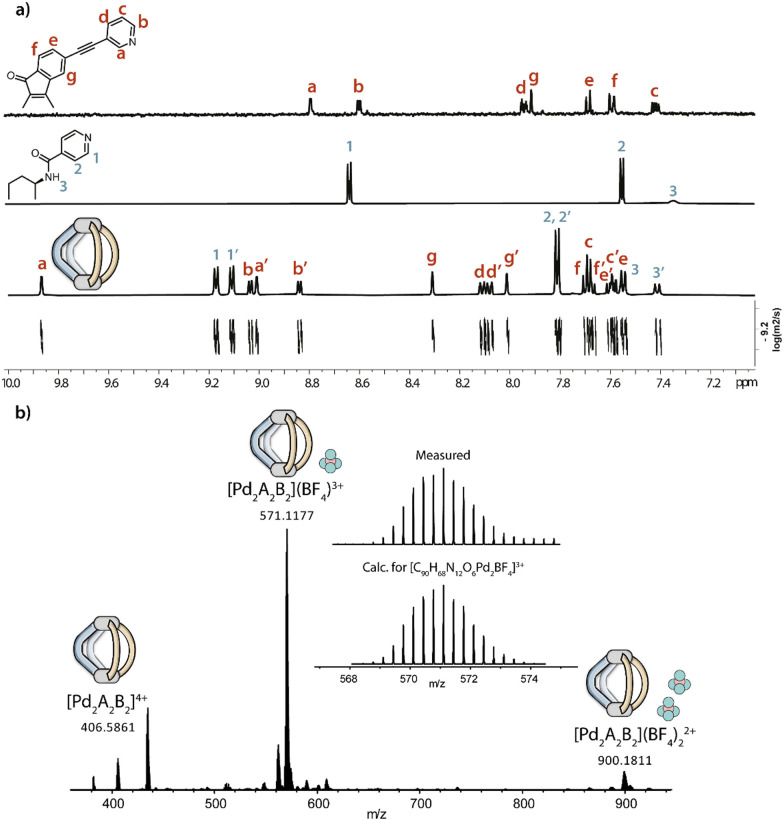
(a) From top to bottom, ^1^H NMR spectra (500 MHz, 298 K, CD_3_CN) of ligand A, ligand B and heteroleptic cage Pd_2_A_2_B_2_, and the DOSY spectrum of Pd_2_A_2_B_2_; (b) ESI-MS spectrum of Pd_2_A_2_B_2_ with the isotopic pattern for [Pd_2_A_2_B_2_ + BF_4_]^3+^ shown in the inset.

Upon assembly, each ^1^H-NMR signal splits into two sets, with the enantiomerically pure B breaking the symmetry of the cage, as the oppositely arranged upper and lower halves of each ligand become non-equivalent. The cage forms as a *cis*-isomer with respect to the ligand arrangement around the square-planar Pd^II^ centres, as evidenced by ROESY cross peaks between protons H_b_–H_b′_, H_a_–H_1′_, H_a′_–H_1_ and H_a_–H_a′_ (Fig. S5, ESI†). The formation of a single species was further confirmed by DOSY-NMR, with all the signals belonging to the same diffusion coefficient (*D* = 6.35 × 10^−10^ m^2^ s^−1^), corresponding to a hydrodynamic radius *r*_H_ = 8.81 Å, in accordance with the formation of a Pd_2_L_2_L′_2_ assembly and a geometry-optimized DFT model (*vide infra*). The assumed stoichiometry was further supported by ESI-MS analysis, showing prominent peaks for the [Pd_2_A_2_B_2_]^4+^ species as well as the analogue 3+ and 2+ charged assemblies with one and two associated BF_4_^−^ counter anions, respectively (Fig. 1b).

Despite several attempts, we were unsuccessful to obtain suitable crystals for X-ray diffraction analysis. To gain further structural insight we prepared a DFT-optimized model (ωb97xd/def2-SVP) of the *cis*-Pd_2_A_2_B_2_ cage (Fig. 2). The model supports the geometric complementarity of the two ligands, connected *via* the square-planar Pd^II^ centres.

**Fig. 2:**
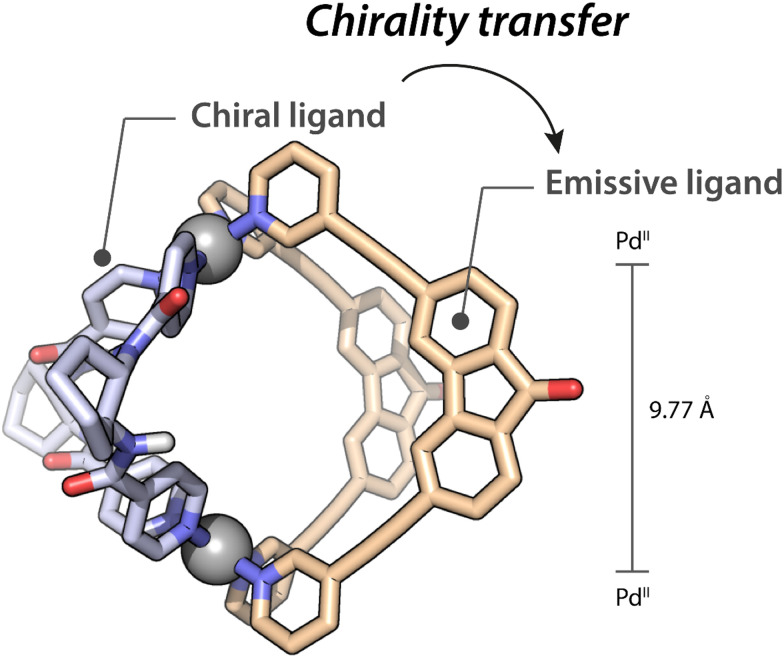
DFT model, side view, of the heteroleptic cage Pd_2_A_2_B_2_. Color code: Pd grey, C beige for A light blue for B, N blue, O red, H white (when not omitted for clarity).

The Pd-pyridine plane is tilted by ≈60° relative to the Pd⋯Pd axis. The short distance between the two pyridine N atoms of B results in a distortion of oppositely arranged ligand A and a rather short Pd⋯Pd distance of 9.8 Å. Interestingly, from a comparison with the previously reported cages,^37^ there appears to be a correlation between the increase of the CPL intensity (as expressed in the *g*_lum_ value) and the observed cage compression (as read from Pd⋯Pd and pyridine N⋯N distances; see ESI†).

After having confirmed the assembly of the heteroleptic cage, we went on to investigate the (chir)optical properties of the system. Chiral ligand B only shows rather weak absorbance below 300 nm, with a broad band centred at 250 nm (Fig. 3). Upon interaction with Pd^II^, the species forms an undefined mixture of compounds (namely Pd_*n*_B_2*n*_), with an absorption spectrum resembling the one of B but decreased optical density, probably due to the formation of larger colloidal aggregates (Fig. S11, ESI†). On the other hand, ligand A, as well as the homoleptic cage Pd_2_A_4_,^37^ shows two absorption maxima at 280 and 300 nm, a series of shoulders between 320 and 360 nm, and a broad band centred at 400 nm. These features are maintained in the absorption spectrum of Pd_2_A_2_B_2_, which therefore resembles the superposition of the absorption spectra of both A and B, with a predominant contribution at *λ* ≥ 275 nm, deriving from the fluorenone moiety.

**Fig. 3:**
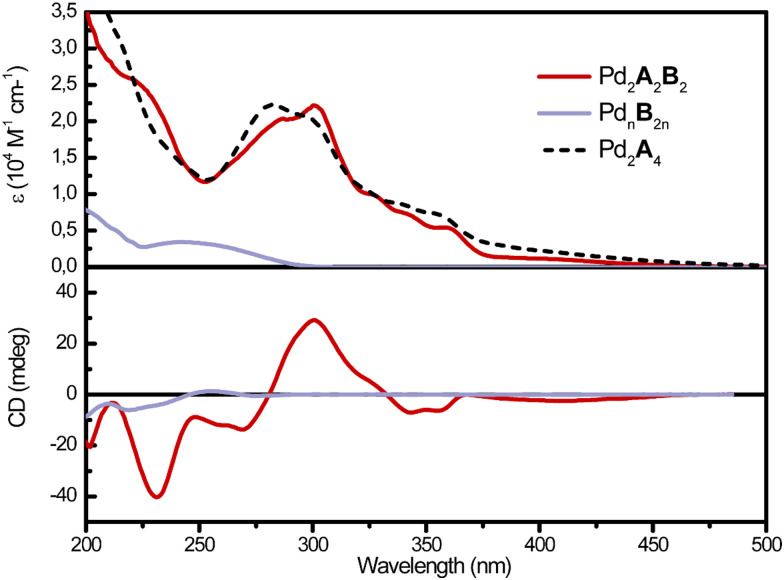
UV-Vis absorption (top) and CD (bottom) spectra of homoleptic species Pd_2_A_4_ (dashed), Pd_*n*_B_2*n*_ (purple), and heteroleptic cage Pd_2_A_2_B_2_ (red; all in CD_3_CN, 0.35 mM with respect to ligand concentration, 298 K).

The first indication of ligand-to-ligand chirality transfer was obtained by CD spectroscopy, showing a strong band at 300 nm, two weaker bands at 340 and 355 nm, and a broader band in the visible range, centred around 400 nm (Fig. 3). In this spectral region, the absorption contribution derives exclusively from the formerly achiral fluorenone chromophore, and thus the presence of a CD signal unambiguously confirms the chirality transfer imparted by B onto the overall assembly and thus the ground state of A (while, by definition, the entire architecture is chiral once a chiral component, here B, is included, the assembly's large size and dynamic nature does not necessarily guarantee locations remote from the stereocenters to be locked in a chiral conformation or experience an immediate chiral environment).

This was further supported by studying the emission properties of the system, in particular its circularly polarized luminescence (CPL). Differently from our previously reported systems,^37^ we here now have the possibility to selectively excite only the emissive ligand A, excluding any concomitant absorption of the chiral ligand. Upon excitation at *λ*_ex_ = 335 nm, the heteroleptic cage was found to clearly exhibit a broad emission centred at 490 nm, ascribed to a π*–π transition localized on the fluorenone backbone, as expected for an aromatic ketone and in accordance with what we previously observed (Fig. 4).^37^ Pleasingly, the heteroleptic cage Pd_2_A_2_B_2_ exhibits CPL in accordance with its fluorescence spectrum (Fig. 4), deriving exclusively from the formerly achiral ligand A and thus further demonstrating a ligand-to-ligand chiral induction. Interestingly, the CPL intensity is almost three times higher compared to our previously reported heteroleptic cage, with the |*g*_lum_| value raising from 9.0 × 10^−4^ for Pd_2_A_2_H_2_ to −2.5 × 10^−3^ for Pd_2_A_2_B_2_.

**Fig. 4:**
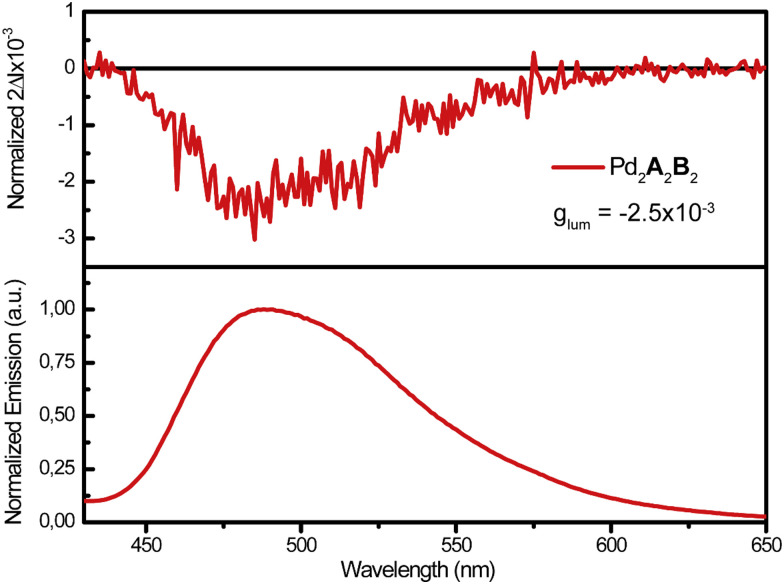
CPL (top) and emission (bottom) spectra of heteroleptic cage Pd_2_A_2_B_2_ (*λ*_ex_ = 335 nm, CD_3_CN, 0.35 mM with respect to ligand concentration, 298 K).

To conclude, we herein report the second example of a Pd^II^-based heteroleptic cage, self-assembled from a chiral and a chromophore-base ligand, showing induced CD and CPL signals deriving exclusively from the electronic transitions of chromophore A that is achiral in its unbound form. These chiroptical properties arise from the synergistic effect of the building blocks by ligand-to-ligand chirality transfer *via* their coordination to the Pd^II^ nodes. The use of the SCA strategy allows us to achieve a high degree of structural and functional complexity, notably with a strongly reduced synthetic effort, compared to the previous examples. The modular character of the heteroleptic cages allowed us to replace helicene-based ligand H with cyclohexane-based ligand B. Furthermore, the absence of overlap between the absorption bands of ligands A and B allows us to clearly connect the ligand-to-ligand chirality transfer to both ground (*via* CD) and excited (*via* CPL) states of the fluorenone-based ligand. Structural distortion of the chromophore by the chiral ligand may be the reason for the observed chiroptical phenomenon. Alternatively, CPL may result from a chiral co-conformation/arrangement of the close pair of luminescent backbones *via* an exciton coupling mechanism. While further studies are required to unravel the mechanistic details, we herein show how a modular approach can be used to improve the overall chiroptical properties of a self-assembled compound, in this case achieving a 3-fold higher CPL intensity than a preceding derivative.

This work was supported by the European Research Council (ERC Consolidator grant 683083, RAMSES) and the Deutsche Forschungsgemeinschaft (DFG, German Research Foundation) under Germany's Excellence Strategy EXC2033, project number 390677874 (“RESOLV”) and GRK2376 (“Confinement Controlled Chemistry”), project number 331085229. The authors thank Dr Kai Wu and Kristina Ebbert for providing ligands and Dr Ananya Baksi for measuring ESI mass spectrometry data.

## Conflicts of interest

There are no conflicts to declare.

## Supplementary Material

CC-059-D3CC00262D-s001
