# Supplementary material for: Modular enhancement of circularly polarized luminescence in Pd2A2B2 heteroleptic cages
Source: Chem Commun (Camb). 2023 Feb 21;59(23):3467–70. doi: 10.1039/d3cc00262d (PMC10019126; doi:10.1039/d3cc00262d)
Supplement: CC-059-D3CC00262D-s001 [file CC-059-D3CC00262D-s001.pdf]

Electronic Supplementary Material (ESI) for ChemComm.
This journal is © The Royal Society of Chemistry 2023

Electronic Supplementary Information

Modular Enhancement of Circularly Polarized Luminescence in $Pd_2A_2B_2$ heteroleptic cages

Jacopo Tessarolo,[†* a,b] Elie Benchimol,[†a] , Abdelaziz Jouaiti,[c] Mir Wais Hosseini,[c] and Guido H. Clever[*a]

a. Department of Chemistry and Chemical Biology, TU Dortmund University, Otto-Hahn-Straße 6, 44227 Dortmund, Germany

b. Present address: Department of Chemistry, Chonnam National University, 77, Yongbong-ro, Buk-gu, Gwangju, 61186, Republic of Korea

c. Laboratoire de Tectonique Moléculaire, UMR Unistra-CNRS 7140, Université de Strasbourg, 4 Rue Blaise Pascal, 67070 Strasbourg, France

* Email: guido.clever@tu-dortmund.de; jacopo@chonnam.ac.kr

† equal contribution

1. Experimental Section 2
1.1 Materials and measurements 2
1.2 Self-assembly of heteroleptic cage $[Pd_2\mathbf{A}_2\mathbf{B}_2](BF_4)_4$ in CD_3CN 2
1.3 Self-assembly of homoleptic mixture $[Pd_n\mathbf{B}_{2n}](BF_4)_{2n}$ in CD_3CN 7
2. Photophysical studies 8
3. Computational studies 10
4. References 14

1. Experimental Section

1.1 Materials and measurements

Unless otherwise stated, all chemicals were obtained from commercial sources and used as received.

Ligands **A**,[1–3] enantiopure **B**,[4,5] and homoleptic cage[1,2] $[Pd_2\mathbf{A}_4]^{4+}$ were prepared according to literature procedures. Gel permeation chromatography (GPC) purification of ligands was performed on a JAI 9210-II NEXT GPC System with a JAIGEL HH-2/HH-1 column combination running with $CHCl_3$ (HPLC grade). High resolution Electrospray Ionization (HR-ESI) mass spectra were recorded on Bruker ESI-timsTOF (electrospray ionization-trapped ion mobility-time of flight) and Compact mass spectrometers. All samples were diluted with spectroscopic grade CH_3CN (1:10) prior to measurement. NMR experiments were measured on Bruker AVANCE III and NEO (500 or 600 MHz) spectrometers. Chemical shifts for ^1H and ^{13}C are reported in ppm with residual solvent as reference: Acetonitrile (1.94 ppm for ^1H, 1.32 ppm for ^{13}C). Abbreviations for signal multiplicity of ^1H-NMR spectra are shown as following: s: singlet, d: doublet, t: triplet, dd: doublet of doublets; dt: doublet of triplets; m: multiplet, br: broad. UV-vis spectra were recorded on a DAD HP-8453 UV-Vis spectrometer. Circular dichroism spectra were recorded in CD_3CN with an Applied Photophysics Chirascan qCD Spectrometer with a temperature-controlled cuvette holder. The spectra were background-corrected and smoothed with a window size of 5. Emission measurements were performed on a Jasco FP-8300 spectrometer and quantum yield determination has been performed on a JASCO ILF-835 integrating sphere as accessory of the JASCO FP-8300. Circularly polarized luminescence measurements were performed using a JASCO CPL-300 spectrophotometer, equipped with a (150 W) Xe lamp as light source. The CPL spectrum of heteroleptic cage $[Pd_2\mathbf{A}_2\mathbf{B}_2](BF_4)_4$ was recorded with an excitation and emission bandwidth of 20 nm, a Digital Integration Time of 4 seconds, a data pitch of 1 nm, and averaged over 10 spectra.

1.2 Self-assembly of heteroleptic cage $[Pd_2\mathbf{A}_2\mathbf{B}_2](BF_4)_4$ in CD_3CN

A 500 µL CD_3CN solution of **A** (250 µL from a 2.8 mM suspension right after sonication, 1.0 eq.) and **B** (250 µL from a 2.8 mM solution, 1.0 eq.) was mixed with $[Pd(CH_3CN)_4](BF_4)_2$ (28.0 µL from a 25 mM stock solution in CD_3CN, 1.0 eq.). Heating the sample at 70 °C overnight afforded a 0.7 mM solution of heteroleptic cage $[Pd_2\mathbf{A}_2\mathbf{B}_2](BF_4)_4$, as confirmed by NMR and ESI-MS analyses.

^{1}H-NMR (500 MHz, 298K, CD_3CN) δ 9.86 (d, J^4 = 1.7 Hz, 2H, Ha), 9.17 (m from a AA' in AA'BB' system, 4H, H1), 9.11 (m from a AA' in AA'BB' system, 4H, H1'), 9.03 (dd, J^3 = 5.9 Hz, J^4 = 1.2 Hz, 2H, Hb), 9.00 (d, J^4 = 1.7 Hz,

2H, Ha’), 8.84 (dd, J^3 = 5.9 Hz, J^4 = 1.2 Hz, 2H, Hb’), 8.31 (s, 2H, Hg), 8.11 (dt, J^3 = 8.3 Hz, J^4 = 1.5 Hz, 2H, Hd’), 8.08 (dt, J^3 = 8.3 Hz, J^4 = 1.5 Hz, 2H, Hd), 8.01 (s, 2H, Hg’), 7.82 (m from a BB’ in AA’BB’ system, 8H, H2 and H2’), 7.68 (m, 6H, overlap of Hf, Hf’ and Hc), 7.59 (m, 4H, overlap of He’ and Hc’), 7.55 (m, 4H, overlap of He and H3), 7.41 (d, J^3 = 8.6 Hz, 2H, H3’), 3.95 (m, 2H, amide-CH’), 3.64 (m, 2H, amide-CH), 2.03, 1.99, 1.81, 1.73, 1.55, 1.34 (series of m, br, CH_2 protons from cyclohexyl, 16H, overlapped with solvent and water signals).

^{13}C NMR (150 MHz, 298K, CD_3CN) δ 192.2, 166.7, 163.1, 156.5, 153.7, 152.8, 151.4, 151.2, 146.7, 146.5, 144.7, 144.6, 144.1, 142.2, 135.4, 135.3, 134.2, 132.9, 129.0, 128.6, 128.5, 128.2, 126.7, 126.6, 125.6, 125.5, 125.4, 125.0, 124.5, 124.3, 95.9, 95.2, 88.2, 87.2, 57.5, 54.0, 32.5, 31.6, 25.5, 25.0.

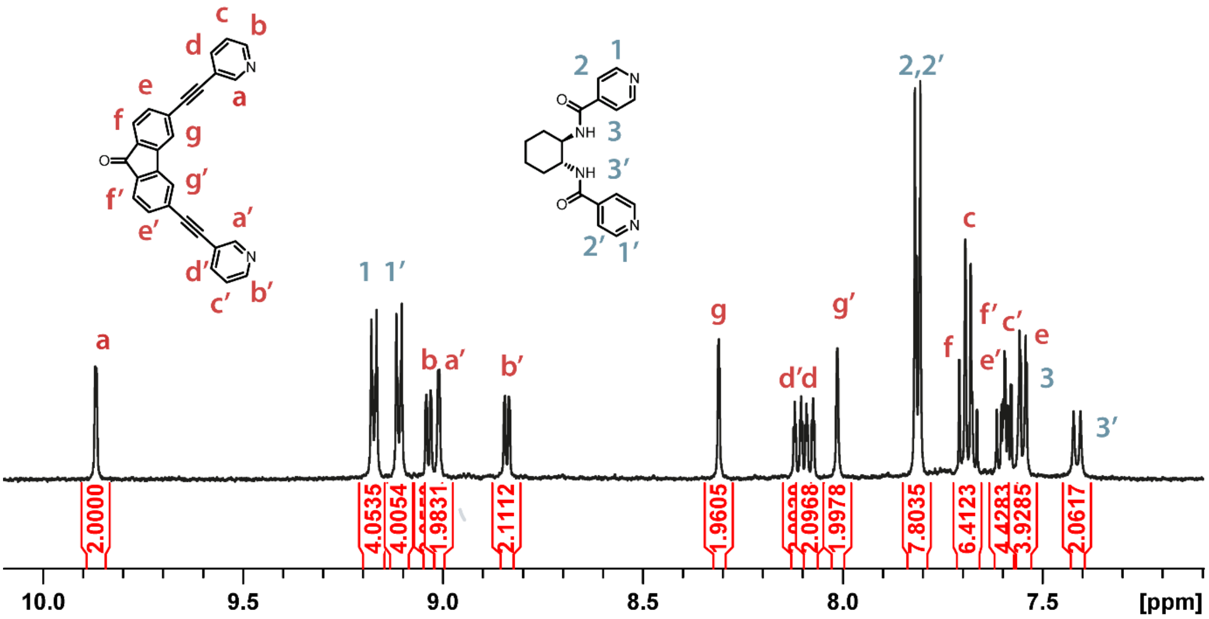

Figure S1. Partial ^{1}H-NMR spectrum (500 MHz, 298K, CD_3CN) of heteroleptic cage $[Pd_2\mathbf{A}_2\mathbf{B}_2](BF_4)_4$.

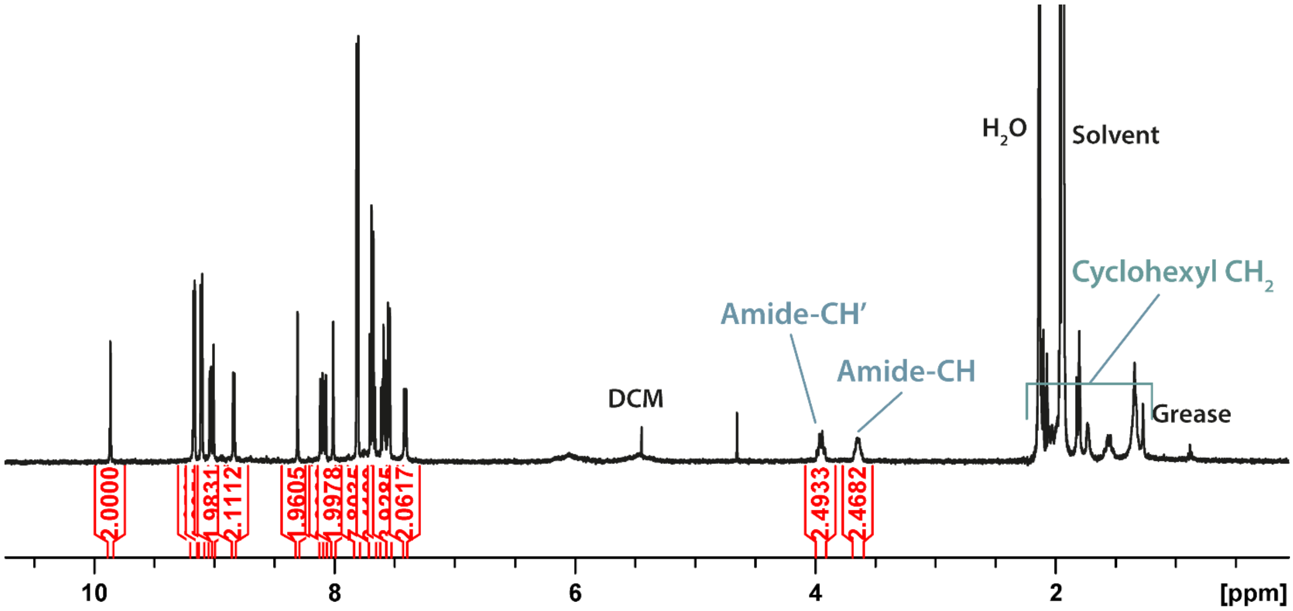

Figure S2. Full ^{1}H-NMR spectrum (500 MHz, 298K, CD_3CN) of heteroleptic cage $[Pd_2\mathbf{A}_2\mathbf{B}_2](BF_4)_4$.

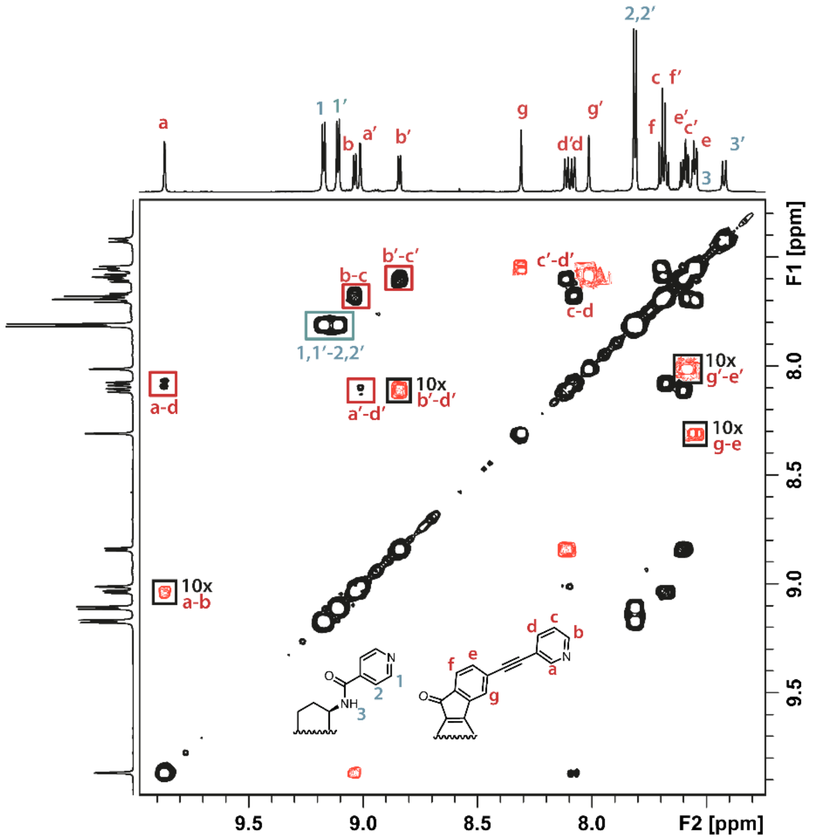

Figure S3. Partial ^{1}H–^{1}H COSY spectrum (600 MHz, 298K, CD_3CN) of heteroleptic cage $[Pd_2\mathbf{A}_2\mathbf{B}_2](BF_4)_4$. Red color: cross peaks zoomed in by a factor of 10.

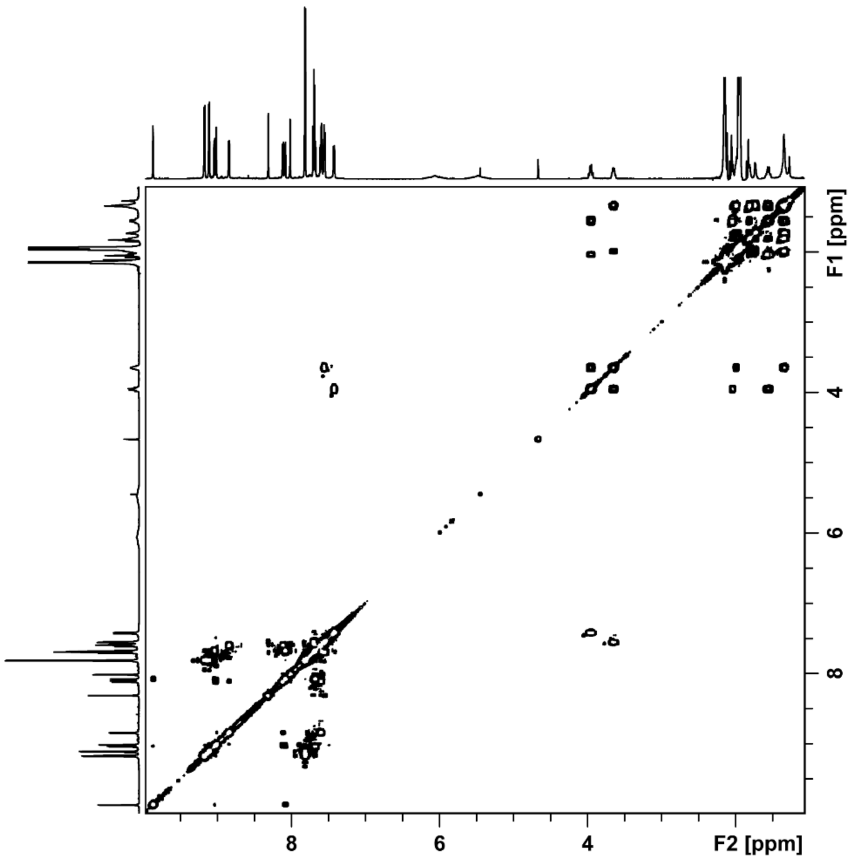

Figure S4. Full ^{1}H–^{1}H COSY spectrum (600 MHz, 298K, CD_3CN) of heteroleptic cage $[Pd_2\mathbf{A}_2\mathbf{B}_2](BF_4)_4$.

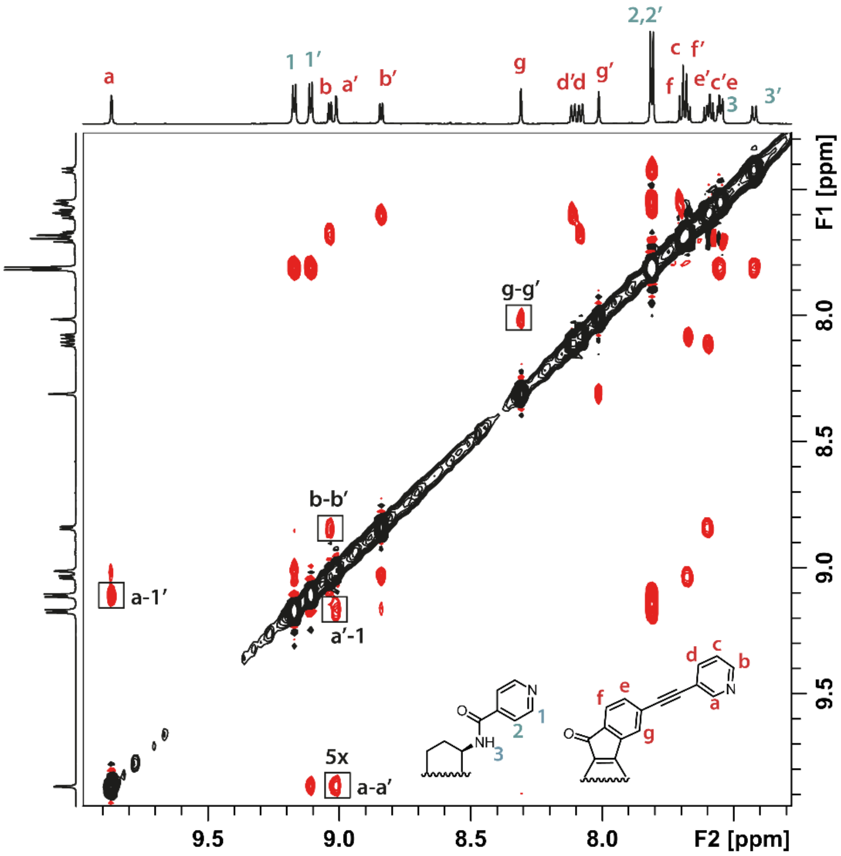

Figure S5. Partial ^{1}H–^{1}H ROESY spectrum (600 MHz, 298K, CD_3CN) of heteroleptic cage [Pd_2**A**$_2$**B**$_2$]$(BF_4)_4$, a-a′ cross peak zoomed in by a factor of 5.

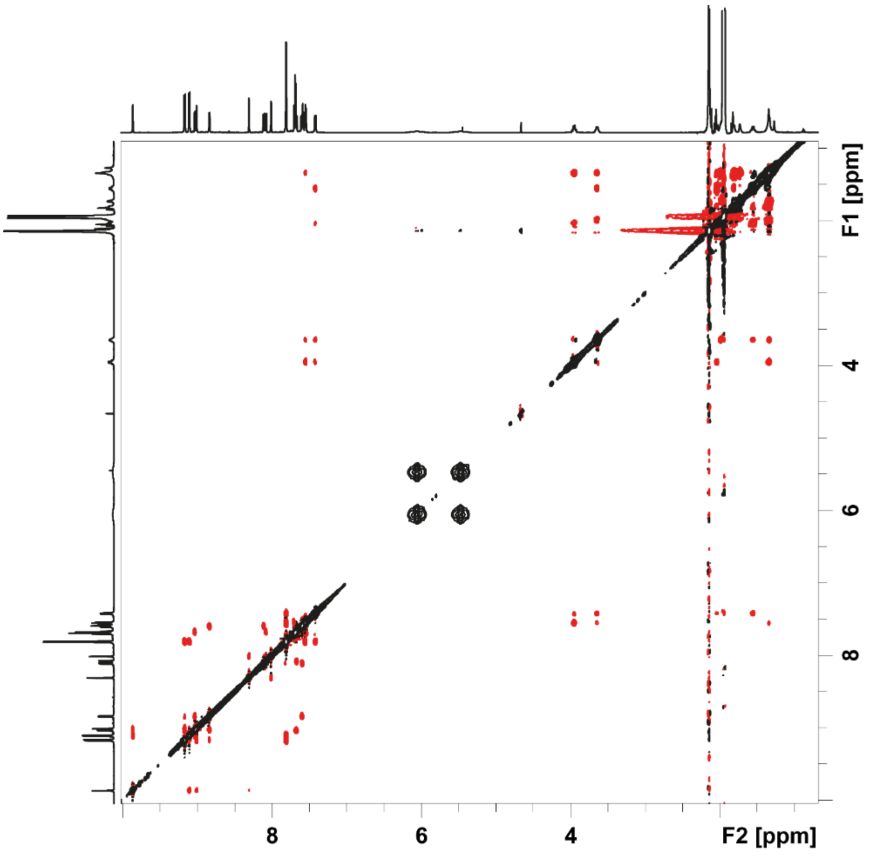

Figure S6. Full ^{1}H–^{1}H ROESY spectrum (600 MHz, 298K, CD_3CN) of heteroleptic cage [Pd_2**A**$_2$**B**$_2$]$(BF_4)_4$.

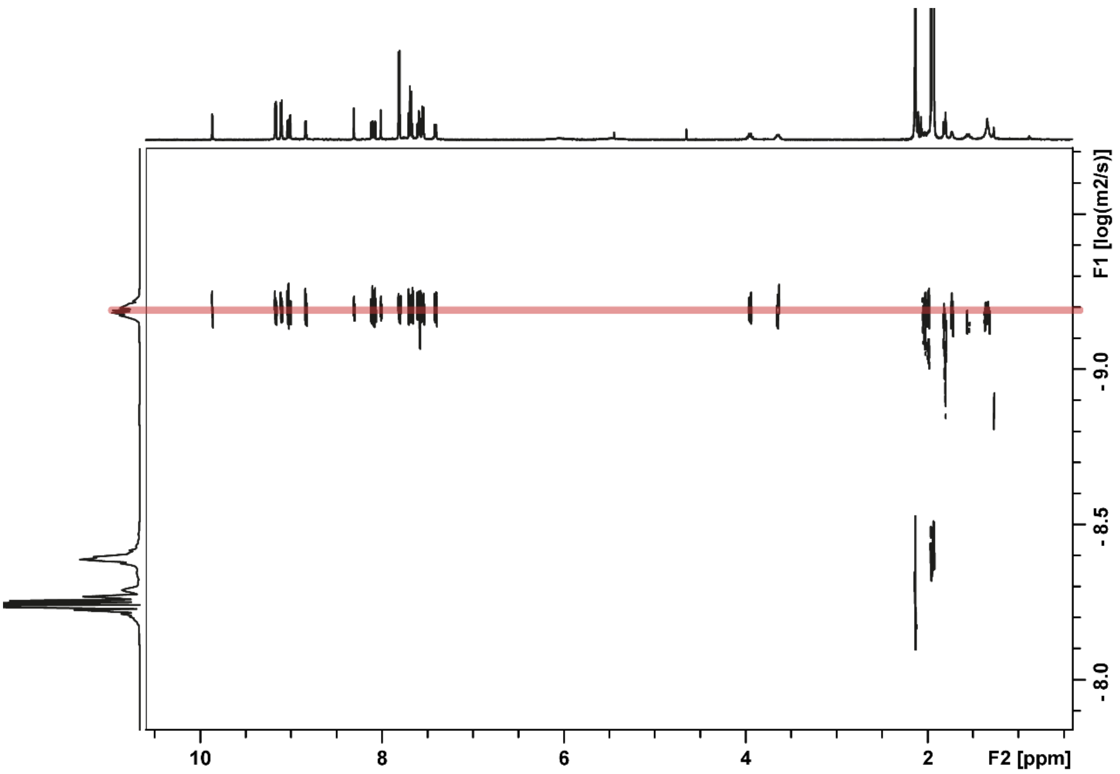

Figure S7. ^{1}H DOSY spectrum (500 MHz, 298K, CD_3CN) of heteroleptic cage $[Pd_2\mathbf{A}_2\mathbf{B}_2](BF_4)_4$. Diffusion coefficient D= 6.35 x 10^{-10} m^2s^{-1}, hydrodynamic radius r_H = 8.81 Å.

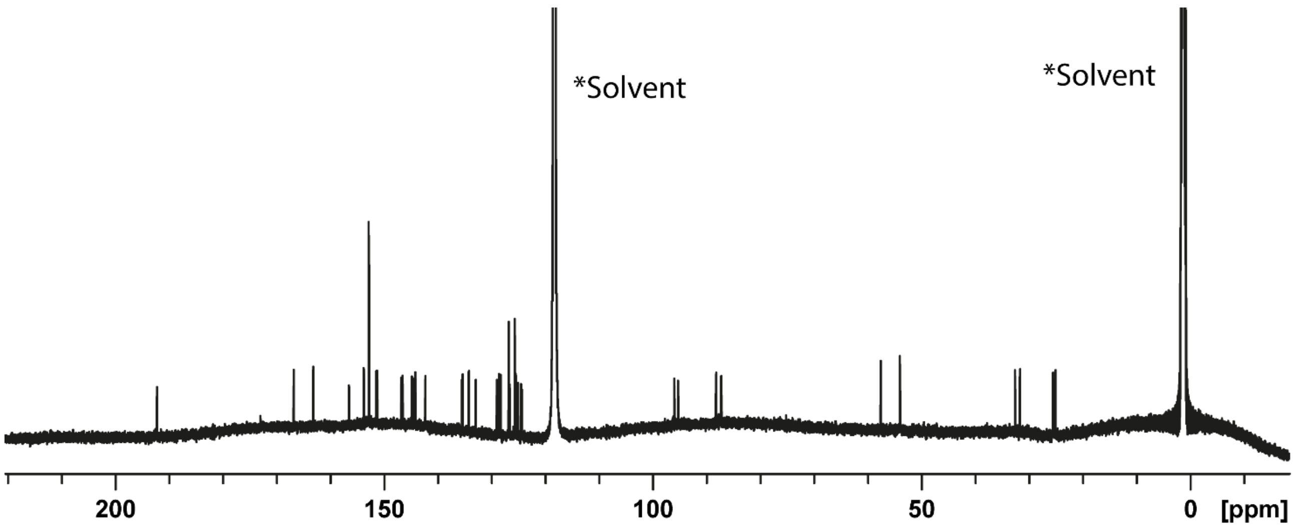

Figure S8. ^{13}C spectrum (150 MHz, 298K, CD_3CN) of heteroleptic cage $[Pd_2\mathbf{A}_2\mathbf{B}_2](BF_4)_4$.

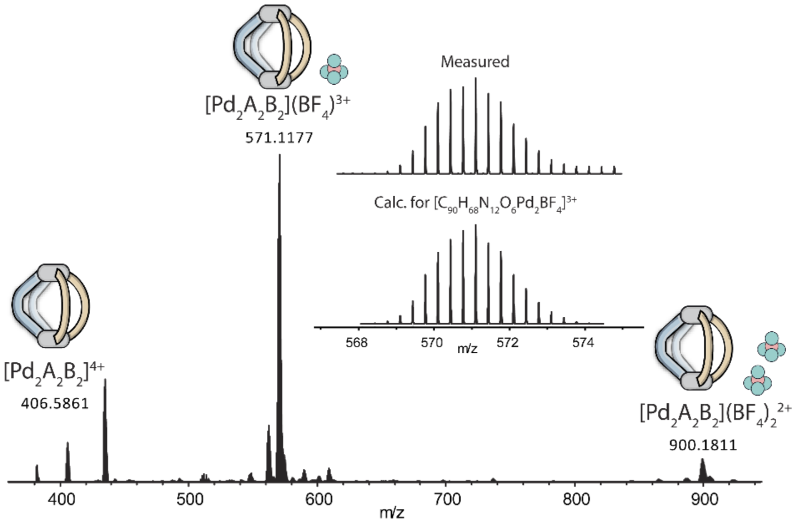

Figure S9. ESI-MS mass spectrum of heteroleptic cage [Pd$_2$**A$_2$B$_2$**](BF_4)$_4$.

1.3 Self-assembly of homoleptic mixture [Pd$_n$**B**$_{2n}$](BF_4)$_{2n}$ in CD_3CN

A solution of [Pd(CH_3CN)$_4$](BF_4)$_2$ (33.6 µL from a 25 mM stock solution in CD_3CN, 0.84 mmol, 1.0 eq.) was added to a solution of ligand **B** (0.54 mg, 1.7 µmol, 2.0 eq.) to give 600 µL of a 2.8 mM CD_3CN solution (expressed in ligand concentration). After heating the sample at 70°C overnight, or up to 4 days, the system resulted in an ill-defined mixture of species with plausible stoichiometry [Pd$_n$**B**$_{2n}$](BF_4)$_{2n}$, as evidenced by ^{1}H-NMR. The upfield shift of protons H1 and H2 supports the coordination of the pyridines to the Pd^{II} cations. Attempts to measure ESI-MS or cryo-ESI-MS of the samples were not successful. Despite the ill-defined character, the sample prepared in this way has been used for the photophysical studies in order to compare the effect of Pd^{II} coordination to the absorption and CD bands of ligand **B**.

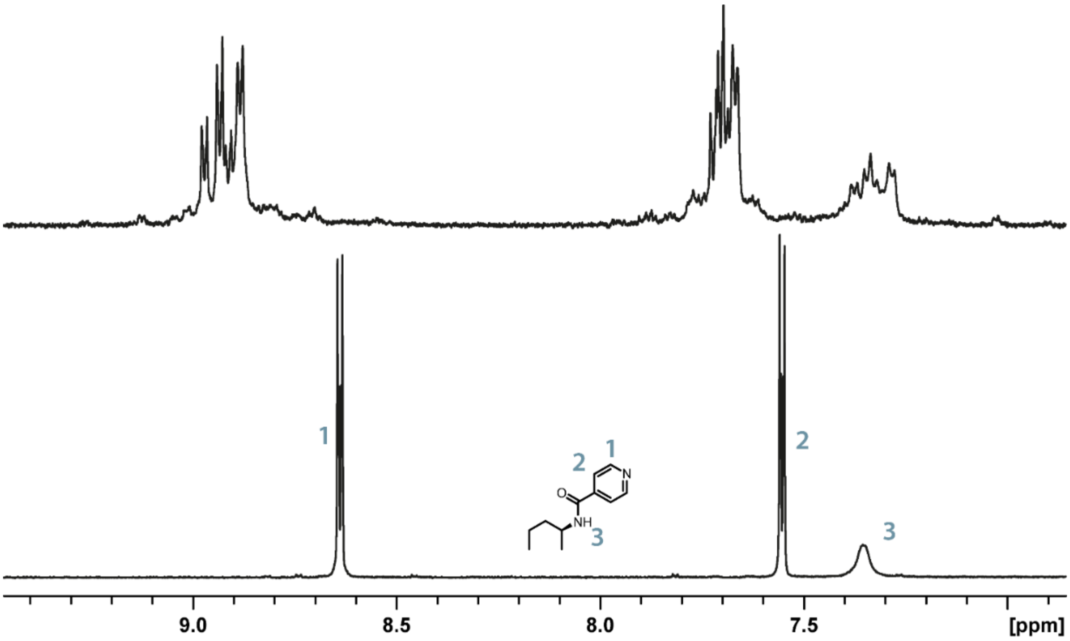

Figure S9. From bottom to top, partial ^{1}H-NMR spectra (500 MHz, 298K, CD_3CN) of ligand **B**, homoleptic mixture [Pd$_n$**B**$_{2n}$](BF_4)$_{2n}$ after heating overnight at 70°C.

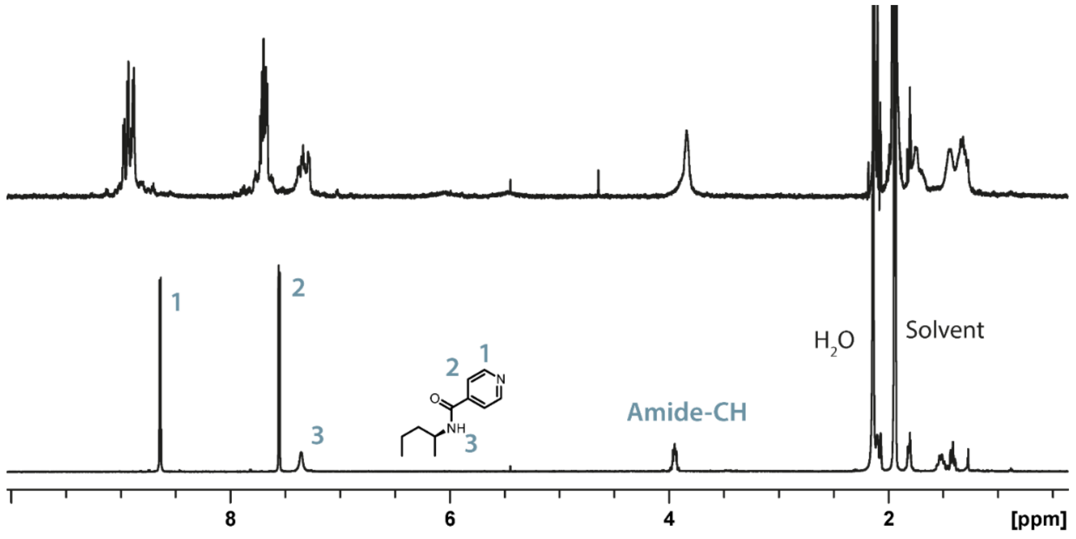

Figure S10. From bottom to top, full ^{1}H-NMR spectra (500 MHz, 298K, CD_3CN) of ligand **B**, homoleptic mixture $[Pd_n\mathbf{B}_{2n}](BF_4)_{2n}$ after heating overnight at 70°C.

2. Photophysical studies

UV-Vis Absorption, CD and CPL measurements were performed using absorption cuvettes with 2 mm path length, a sample concentration of 3.5×10^{-4} M expressed as ligand concentration, and CD_3CN as solvent. The spectra of ligand **A** and homoleptic $[Pd_2\mathbf{A}_4](BF_4)_4$ are taken from the literature[2] and here reported again for better comparison.

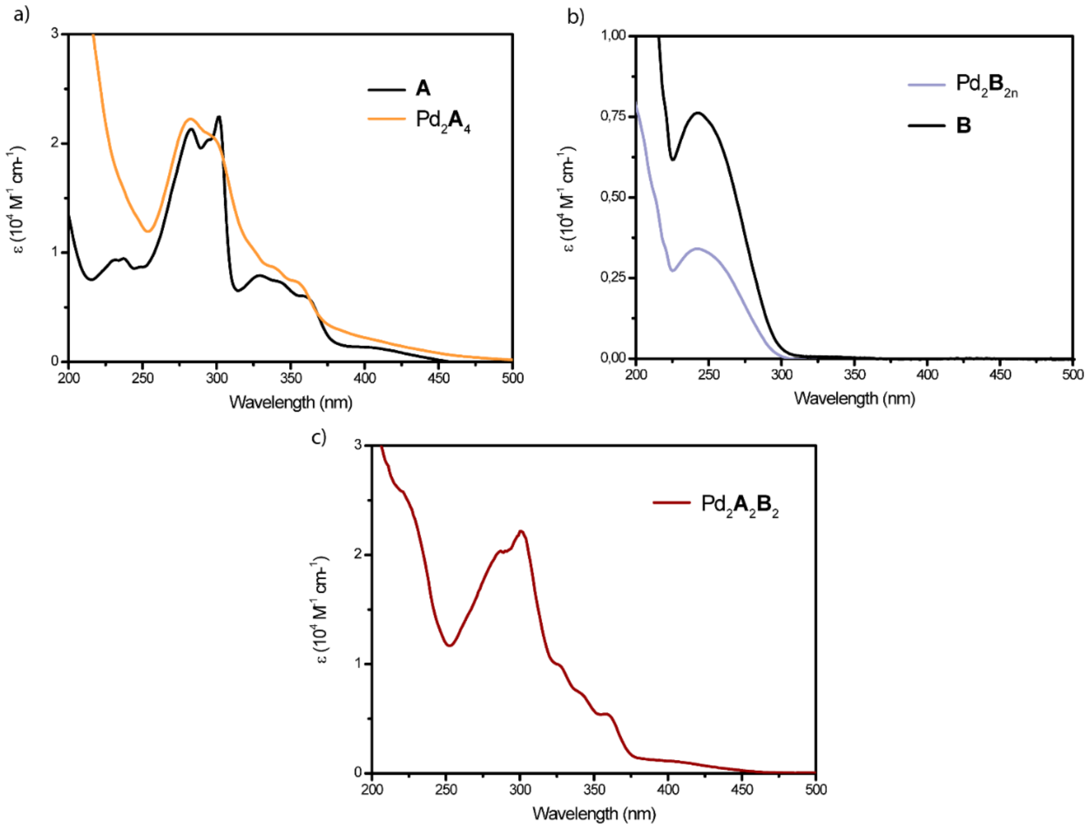

Figure S11. UV-Vis spectra of a) ligand **A** and $[Pd_2\mathbf{A}_4](BF_4)_2$ as previously reported;[2] b) ligand **B** and homoleptic $[Pd_n\mathbf{B}_{2n}](BF_4)_{2n}$, and c) heteroleptic cage $[Pd_2\mathbf{A}_2\mathbf{B}_2](BF_4)_4$ (CD_3CN, RT, 3.5×10^{-4} M expressed as ligand concentration).

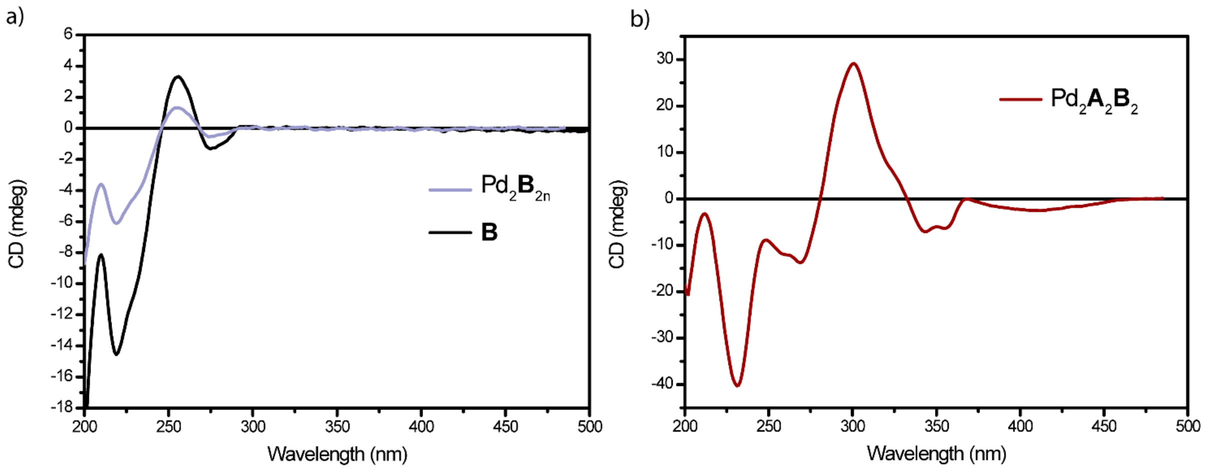

Figure S12. CD spectra of a) ligand **B** and homoleptic $[Pd_n\mathbf{B}_{2n}](BF_4)_{2n}$, and b) heteroleptic cage $[Pd_2\mathbf{A}_2\mathbf{B}_2](BF_4)_4$ (CD_3CN, RT, 3.5×10^{-4} M expressed as ligand concentration).

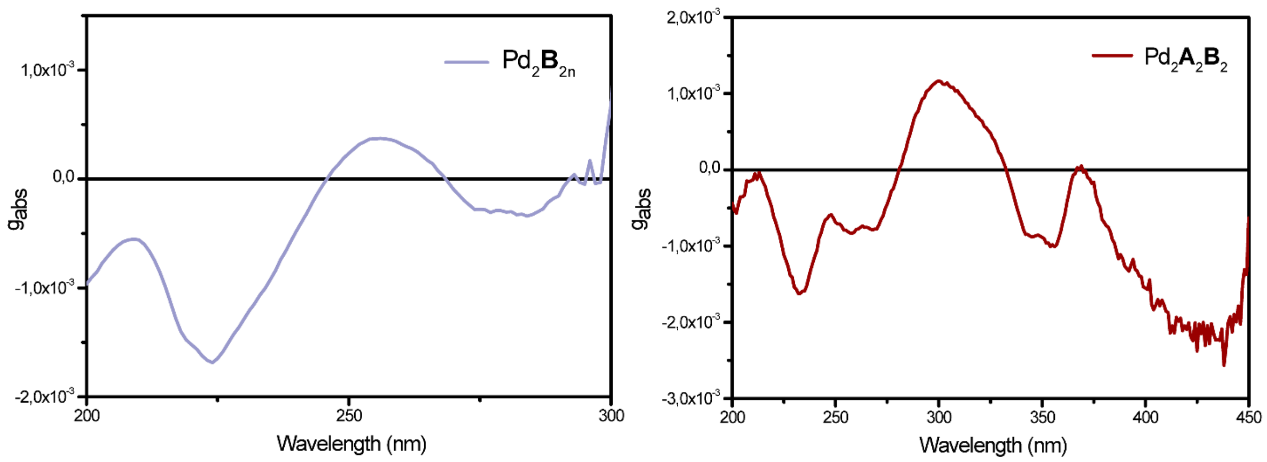

Figure S13. g_{abs} spectra of homoleptic $[Pd_n\mathbf{B}_{2n}](BF_4)_{2n}$ and heteroleptic cage $[Pd_2\mathbf{A}_2\mathbf{B}_2](BF_4)_2$ (CD_3CN, RT, 3.5×10^{-4} M expressed as ligand concentration).

Table S1. Selected g_{abs} values

Cage	g_{abs} (nm)
$Pd_2\mathbf{A}_2\mathbf{B}_2$	$-2 \cdot 10^{-3}$ (425 nm); $-0.9 \cdot 10^{-3}$ (350 nm); $1.1 \cdot 10^{-3}$ (300 nm); $-1.6 \cdot 10^{-3}$ (232 nm)
$Pd_n\mathbf{B}_{2n}$	$-3.4 \cdot 10^{-4}$ (284 nm); $3.7 \cdot 10^{-4}$ (256 nm); $-1.6 \cdot 10^{-3}$ (224 nm)

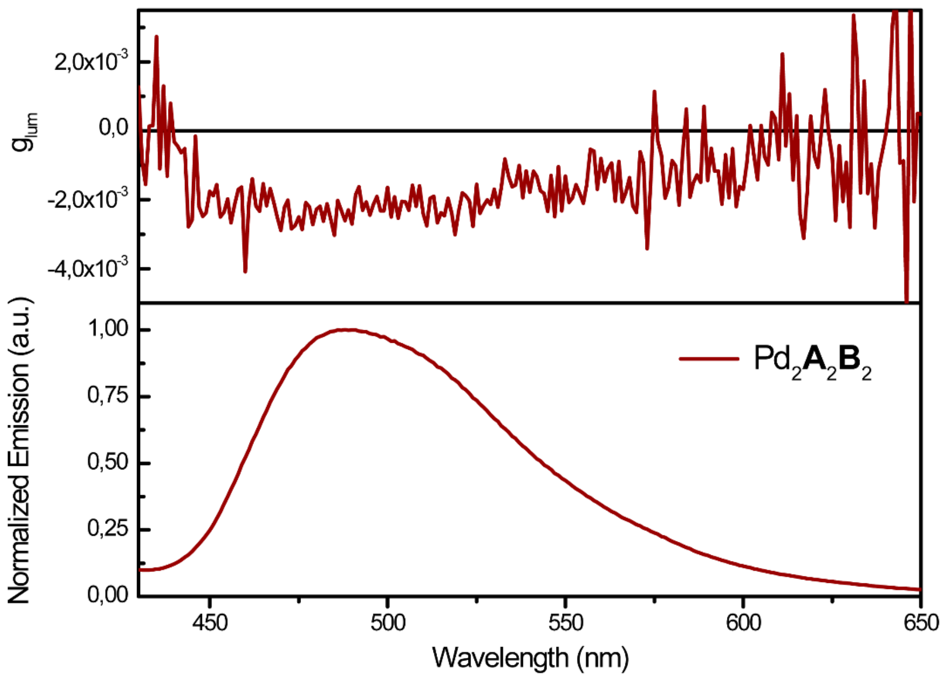

Figure S14. From top to bottom, g_{lum} and normalized emission spectra of heteroleptic cage [Pd_2**A**$_2$**B**$_2$](BF_4)$_4$ (CD_3CN, RT, 3.5x10^{-4} M expressed as ligand concentration). For CPL spectrum see Fig. 4 in main text.

Table S2. Quantum yield determination

	Pd_2**A**$_2$**B**$_2$
**QY** (%)	1.65

λ_{ex} = 335 nm, CD_3CN, RT, concentrations chosen that the absorption at the excitation wavelength does not exceed 0.1, 3 mm quartz cuvette. Measured on a JASCO ILF-835 integrating sphere as accessory of the JASCO FP-8300 spectrofluorometer. 4 measurements were made and averaged.

3. Computational studies

A gas-phase DFT geometry optimization was performed including dispersion correction, using the ωb97xd/def2-SVP level of theory, and computed using the Gaussian 16 software.[6] The obtained structure of [Pd_2**A**$_2$**B**$_2$]$^{4+}$ was then compared with the data from the previously reported helicene-based heteroleptic cages [Pd_2**A**$_2$**H**$_2$]$^{4+}$ and [Pd_2**C**$_2$**H**$_2$]$^{4+}$, where **H** is an helicene-ligand as the *M* enantiomer and **C** is a fluorenone based ligand, where the alkyne linkers are replaced by 1,4-phenyl linkers (Table S1).[2] Interestingly, it is possible to notice a trend between the increase of the observed g_{lum} value and the decrease of the Pd···Pd distances and the N···N distances between the two pyridines of each of the fluorophore-containing ligands. The same applies to the C=O···C=O distances among the two fluorenone backbones in each heteroleptic cage. These values have been chosen to describe both the distortion imparted by the chiral ligand onto the fluorophore ligand, as well as the relative distance between the two chromophores. Owing to structural flexibility in the case of [Pd_2**A**$_2$**B**$_2$]$^{4+}$, there are two different distances given for the N···N distances of the fluorophore-containing ligands.

Furthermore, frontier orbital analysis of the ground state geometry shows that the HOMO-LUMO transition is – as expected for an aromatic ketone – between a π-MO delocalized on the fluorenone aromatic backbone system, featuring a nodal plane concerning the C=O moiety (Fig S15 left), and a π*-MO spanning the aromatic rings and C=O group (Fig S15 right), in full agreement with the previously reported data.[2]

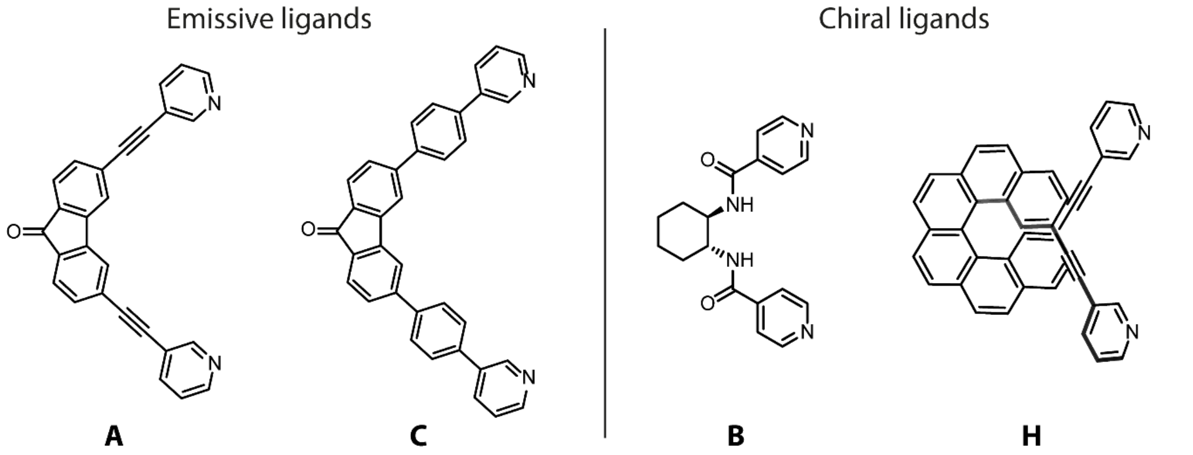

Scheme S1. Emissive and Chiral ligands used to form $Pd_2L_2L'_2$ heteroleptic cages with the name used in the following structural analysis.

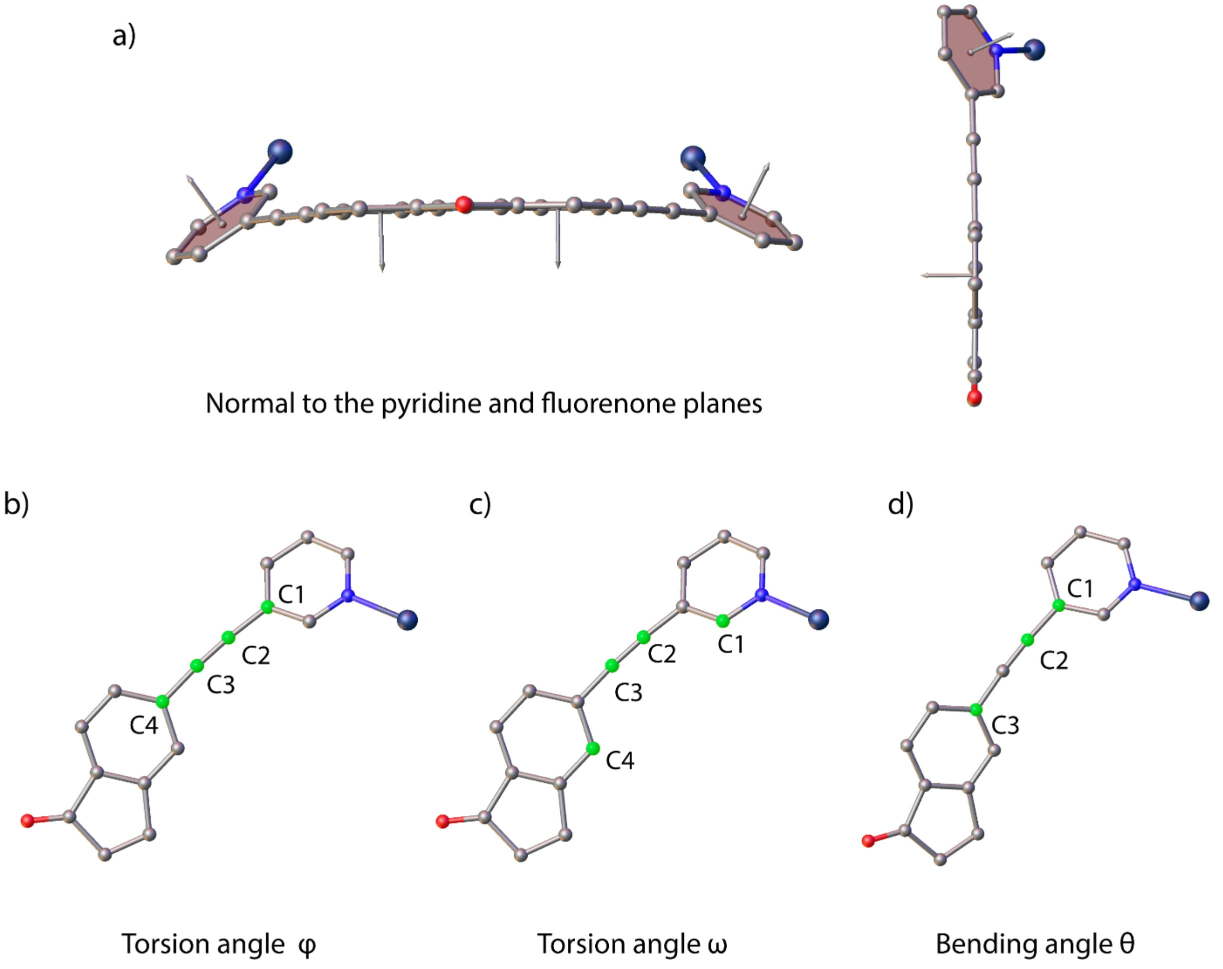

Figure S15. Depiction of structural parameters under investigation in ligand **A** and cages containing ligand **A**: a) normals to the pyridine and fluorenone planes (horizontal and vertical views, respectively); b) dihedral angle φ along the C≡C bond, c) dihedral angle ω highlighting the torsion between the pyridine and fluorenone planes and d) bending angle θ for the C≡C bond. Values see Table S3.

Table S3. Structural parameters of DFT-optimized (ωb97xd/def2-SVP) cages in comparison

**Cage→**	$Pd_2\mathbf{A}_2\mathbf{B}_2$	$Pd_2\mathbf{A}_2\mathbf{H}_2$	$Pd_2\mathbf{C}_2\mathbf{H}_2$
Pd···Pd d (Å)	9.78	12.32	13.63
Pyridine's N···N d (Å)<br>From ligands **A** or **C**	11.57 / 11.31	13.16	14.81
C=O···C=O d (Å)<br>From ligands **A** or **C** backbones	13.14	14.69	16.87
Pyridine's N···N d (Å)<br>From ligands **B** or **H**	7.90 / 8.19	11.60	12.53
Angles between the normals to the pyridine and fluorenone planes in **A** (°)[a]	149.72 / 143.39<br>15.78 / 4.48	155.13<br>7.80	-
dihedral angles φ (°)[a] in **A**	0.43 / 6.53<br>3.09 / -6.79	38.56<br>5.15	-
dihedral angle ω (°)[a] in **A**	27.94/ -32.80<br>-14.09 / 4.50	24.68<br>8.01	-
bending angle θ (°)[a] in **A**	175.56 / 177.68<br>175.44 / 173.52	176.47<br>178.67	-
$\|g_{lum}\|$	-2.5×10^{-3}	0.9×10^{-3}	0.4×10^{-3}

[a] Per distinguishable ligand, two angles are given. Further, the two ligands **A** in $Pd_2\mathbf{A}_2\mathbf{B}_2$ are non-equivalent in the converged DFT-optimized geometry due to the pronounced flexibility of **B**, leading to four distinguishable angles.

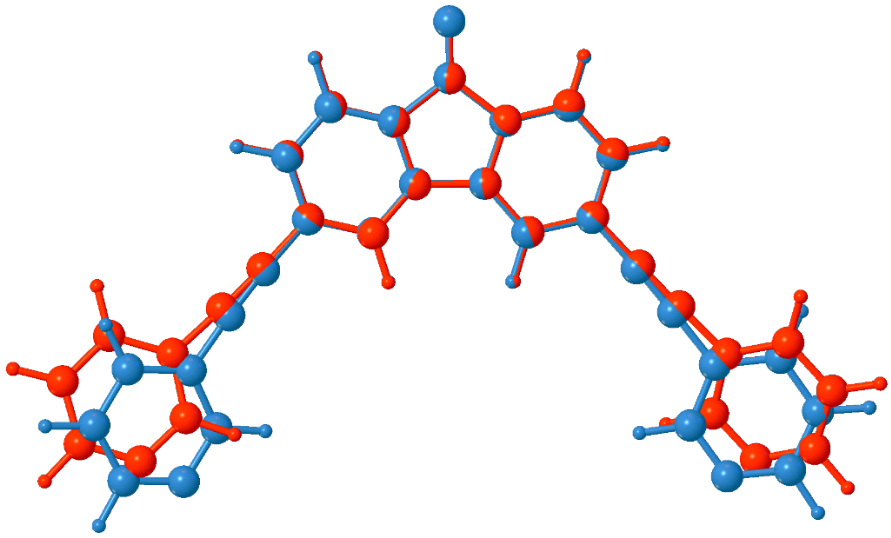

Figure S16. Overlay between ligands **A** cut from the ωb97xd/def2-SVP geometry optimization of $Pd_2\mathbf{A}_2\mathbf{B}_2$ (cyan) and $Pd_2\mathbf{A}_2\mathbf{H}_2$ (orange).[2]

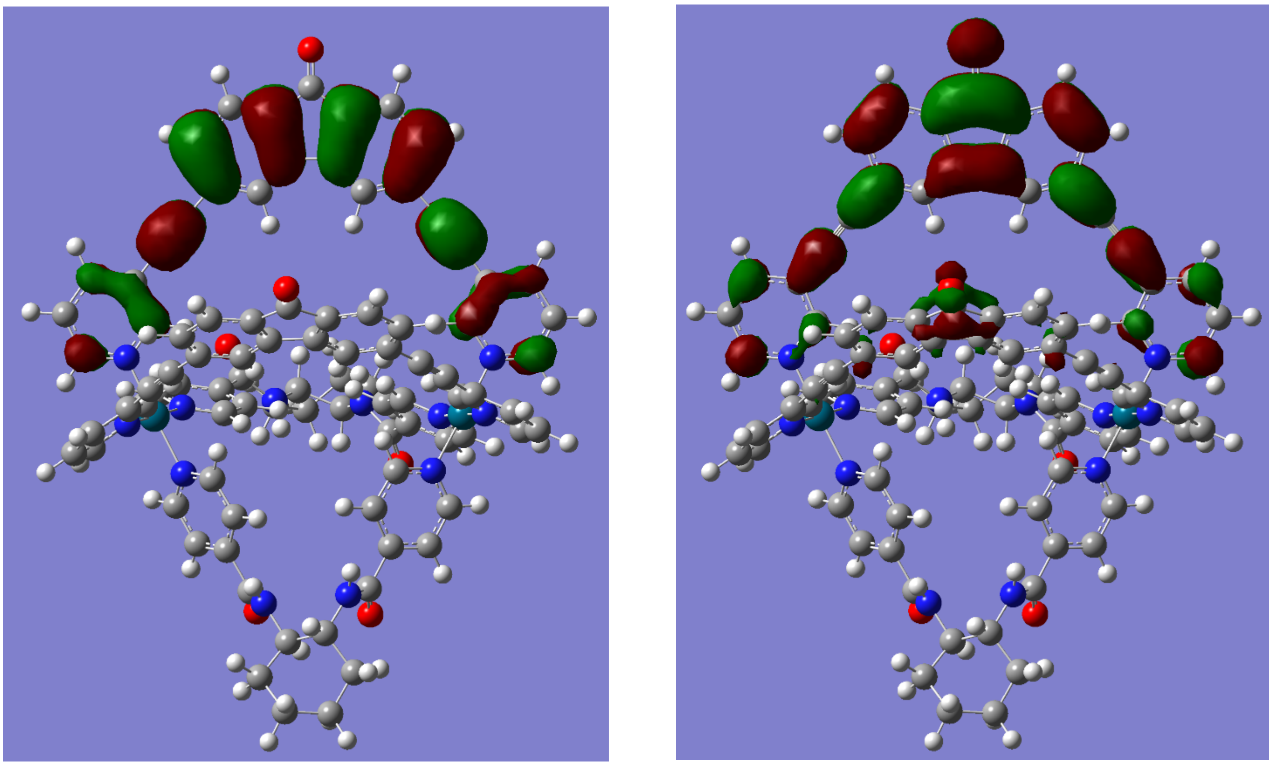

Figure S17. Geometry-optimized ground state structure of $[Pd_2\mathbf{A}_2\mathbf{B}_2]^{4+}$ (ωb97xd/def2-SVP; Pd: purple, C: grey, O: red, N: blue, H: white). Left: HOMO, right: LUMO.

4. References

1. Ebbert, K.E., Schneider, L., Platzek, A., Drechsler, C., Chen, B., Rudolf, R., and Clever, G.H., Resolution of minor size differences in a family of heteroleptic coordination cages by trapped ion mobility ESI-MS. Dalton Trans., 2019, 48, 11070-11075, 10.1039/c9dt01814j.

2. Wu, K., Tessarolo, J., Baksi, A., and Clever, G.H., Guest-modulated Circularly Polarized Luminescence by Ligand-to-Ligand Chirality Transfer in Heteroleptic Pd(II) Coordination Cages. Angew. Chem., Int Ed., 2022, 61, e2022057, 10.1002/anie.202205725.

3. Croué, V., Krykun, S., Allain, M., Morille, Y., Aubriet, F., Carré, V., Voitenko, Z., Goeb, S., and Sallé, M., A self-assembled M2L4 cage incorporating electron-rich 9-(1,3-dithiol-2-ylidene)fluorene units. New J. Chem., 2017, *41*, 3238–3241. 10.1039/c7nj00062f.

4. Chen, H., An, H., Liu, X., Wang, H., Chen, Z., Zhang, H., and Hu, Y., A host–guest hybrid framework with Anderson anions as template: synthesis, crystal structure and photocatalytic properties. Inorg. Chem. Commun., 2012, *21*, 65–68. 10.1016/j.inoche.2012.04.014.

5. Chen, Y., Ding, Y., He, S., Huang, C., Chen, D., and Zhu, B., Synthesis, crystal structures and vapor adsorption properties of mercury(II) coordination polymers derived from two dipyridylamide ligands. Z. Anorg. Allg. Chem., 2021, *647*, 623–628. 10.1002/zaac.202000346.

6. Frisch, M.J., Trucks, G.W., Schlegel, H.B., Scuseria, G.E., Robb, M.A., Cheeseman, J.R., Scalmani, G., Barone, V., Petersson, G.A., Nakatsuji, H., et al. (2016). Gaussian 16, Revision C.01 (Gaussian, Inc.).
